# Unravelling behavioural contributions to IBS risk: evidence from univariate and multivariate Mendelian randomisation

**DOI:** 10.7189/jogh.15.04112

**Published:** 2025-04-11

**Authors:** Hongyu Chu, Yumin Zhong, Jiayi Zhao, Yuezhan Shan, Xuedong Fang

**Affiliations:** Department of Gastrointestinal and Colorectal Surgery, China-Japan Union Hospital of Jilin University, Changchun, China

## Abstract

**Background:**

While numerous studies have investigated the link between behavioural factors and irritable bowel syndrome (IBS), the causal relationships remain unresolved. This study applied Mendelian randomisation (MR) analysis to assess the causal impact of specific behavioural factors on IBS risk.

**Methods:**

Bidirectional Mendelian randomisation analysis was employed to evaluate the causal relationships between behavioural factors and IBS risk. A genome-wide significance threshold (*P* < 5e^−6^) was applied to identify associations between genetic variants and behaviour-related traits, ensuring robust selection of instrumental variables for evaluating potential causal effects. Genetic correlations with IBS were sourced from extensive genome-wide association studies (GWASs). Various statistical methods were applied to estimate the causal effects.

**Results:**

This study employed both univariate and multivariate Mendelian randomisation analyses to investigate the causal relationships between specific behavioural factors and the risk of irritable bowel syndrome (IBS). The results indicated that body mass index (BMI) (odds ratio (OR) = 1.074; 95% confidence interval (CI) = 1.025–1.125, *P* = 0.031), insomnia (OR = 1.986; 95% CI = 1.652–2.389, *P* < 0.001), duration of mobile phone use (OR = 1.120; 95% CI = 1.018–1.232, *P* = 0.021), and weekly mobile phone usage time in the past three months (OR = 1.148; 95% CI = 1.016–1.298, *P* = 0.021,) were associated with an increased risk of IBS. In contrast, usual walking speed (OR = 0.756; 95% CI = 0.621–0.920, *P* < 0.001), non-smoking status (OR = 0.779; 95% CI = 0.645–0.941, *P* < 0.001), and weekly alcohol consumption (OR = 0.862; 95% CI = 0.743–0.999, *P* = 0.015) were associated with a reduced risk of IBS. Furthermore, in the multivariate Mendelian randomisation analysis, no statistically significant causal associations were found for BMI, usual walking pace, length of mobile phone use, and smoking status. Weekly mobile phone usage time in the past three months (OR = 1.439; 95% CI = 1.126–1.840, *P* = 0.0037,) and insomnia (OR = 1.468; 95% CI = 1.076–2.003, *P* = 0.0156) were identified as risk factors, while weekly alcohol intake (OR = 0.813; 95% CI = 0.677–0.975, *P* = 0.0257) acted as a protective factor.

**Conclusions:**

This study identified BMI, insomnia, duration of mobile phone use, and weekly mobile phone usage time in the past three months as risk factors for IBS. In contrast, weekly alcohol consumption, usual walking pace, and non-smoking status were observed as protective factors. Additionally, in multivariable analysis, weekly mobile phone use, insomnia, and weekly alcohol consumption showed a direct influence on IBS risk when considered simultaneously.

Irritable bowel syndrome (IBS) is a digestive disorder marked by symptoms such as abdominal pain, bloating, and irregular bowel habits. This disorder occurs without any apparent signs of organic intestinal disease [1,2]. It is estimated to have a global prevalence of 10 to 20%, with affected individuals experiencing significant economic burdens and reduced quality of life [3]. There is a growing recognition of the importance of patient-centred clinical data on the impact of IBS to better prevent the disease and enable clinicians, health care systems, funding agencies, and regulators to better treat patients.

An individual's genetics, biological make-up, and environment may all play a role in IBS. Factors such as smoking, alcohol intake, and elevated body mass index (BMI) may contribute to the onset of IBS, with these behaviours potentially playing a substantial role in its development [4]. Notably, individuals who smoke heavily have an elevated risk of developing diarrhoea-predominant IBS compared to non-smokers. However, research by Choung et al. found no significant correlation between IBS and BMI [5]. Sleep quality also plays a crucial role, with healthy sleep patterns linked to a reduced risk of IBS [6]. Most of these findings stem from observational studies, which are limited by design constraints, sample size, and confounding variables, thus precluding definitive conclusions. In addition, in recent years, with the rapid development of technology, the use of mobile phones has increased significantly, and studies have found a potential association between screen viewing time and cancer and other adverse health outcomes [7–9]. Previous research has indicated that exercise can alleviate IBS symptoms; for instance, six weeks of treadmill aerobic exercise improves the quality of life and reduces symptom severity [10]. However, the impact of normal walking speed on IBS remains unexplored. Based on these findings, we selected these factors as exposure factors to explore their potential causal association with the onset of IBS.

Mendelian randomisation (MR) is commonly employed as a method to evaluate causal relationships in disease research [11]. The MR model is based on the second law of Mendel, which states that one trait should inherit independently of other traits. This is similar to a random process at conception. This allows for ‘natural’ randomised controlled trials (RCTs). As compared to traditional RCTs, MR has the advantage of overcoming confounders and reversing causality [12]. In bidirectional two-sample MR studies, gene-exposure and gene-outcome data are collected from two independent samples drawn from the same population. This approach is more economical and efficient, and bidirectional MR can help disentangle these relationships [13].

This study employed both univariable MR (UVMR) and multivariable MR (MVMR) methods to explore causal links between several behavioural factors and IBS, using single nucleotide polymorphism (SNP) data from public databases. Sensitivity analyses were conducted to enhance the robustness of the findings by assessing how key assumptions influenced the results.

## METHODS

**Figure 1:**
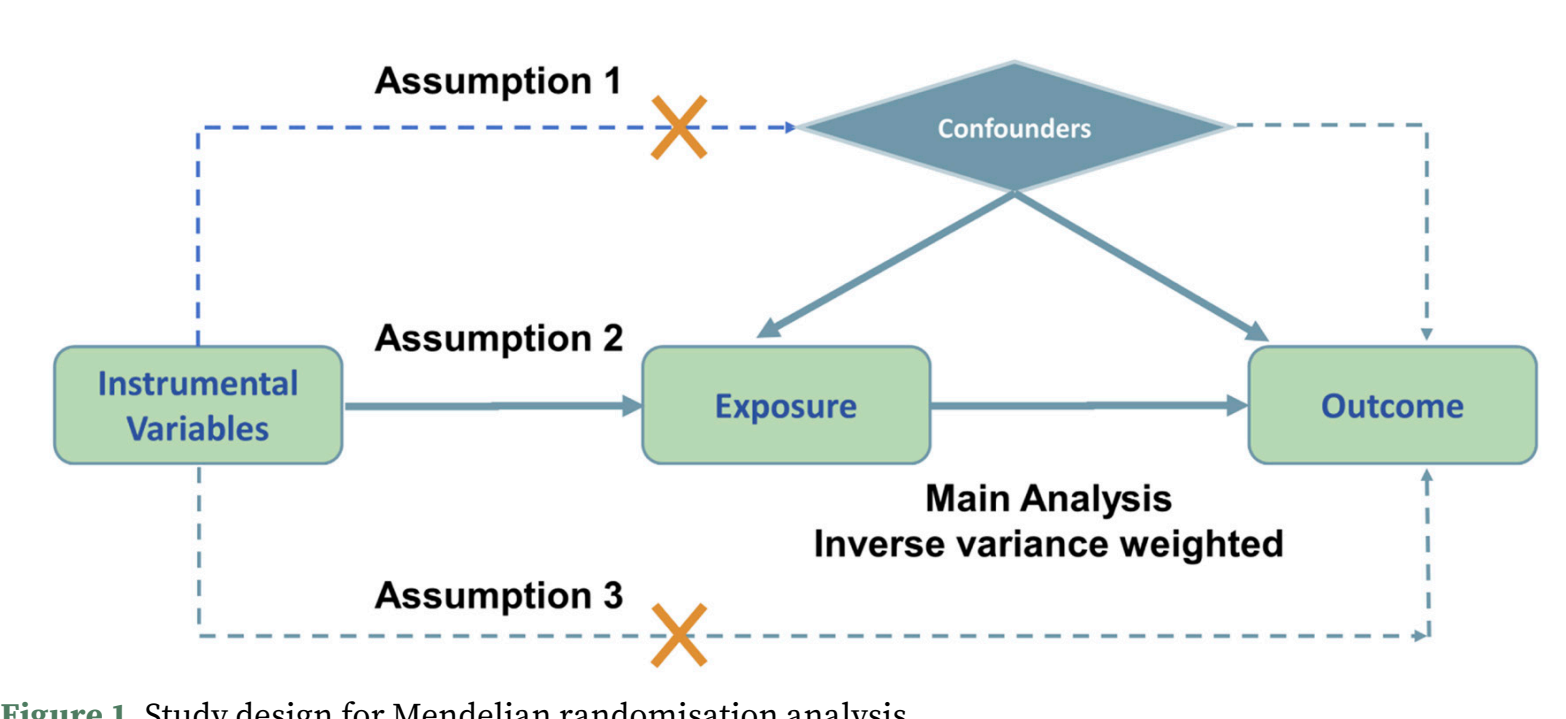
Study design for Mendelian randomisation analysis.

For this MR analysis, genetic variants linked to seven behavioural factors or IBS were extracted from GWAS to function as instrumental variables (IVs). To qualify as IVs, these genetic variants needed to meet three key criteria (**Figure 1**):

1) a strong link with the exposure

2) independence from confounding variables affecting the exposure-outcome relationship

3) an impact on the outcome exclusively through the exposure [1,2,14]. Data sets were retrieved from the Integrative Epidemiology Unit Open Genome-Wide Association Study (IEU Open GWAS) database (**Table 1**).

**Table 1:** Overview of genome-wide association study (GWAS) data sets utilised in this study

GWAS ID	Trait	Definition	Author (year)	Sample size		Number of SNPs	Population
ukb-a-248	BMI	Weight divided by the square of height, expressed in kilograms per square meter	Neale (2017)		336 107	10 894 596	European
ukb-a-513	Usual walking pace	The habitual walking speed of individuals in daily life	Neale (2017)		335 349	10 894 596	European
ukb-b-3957	Insomnia	Participants report whether they have difficulty falling asleep at night or waking up in the middle of the night	Ben Elsworth (2018)		462 341	9 851 867	European
ukb-b-4094	Length of mobile phone use	How many years have participants reported using their mobile phones to make or receive calls at least once a week	Ben Elsworth (2018)		456 972	9 851 867	European
ukb-b-17999	Weekly usage of mobile phone in last 3 months	Participants reported how much time they spent on their phones on average per week in the past 3 months	Ben Elsworth (2018)		386 626	9 851 867	European
ukb-d-20116_0	Smoking status: Never	Smoking status of participants: Yes or No	Neale laboratory (2018)		359 706	13 586 591	European
ieu-b-73	Alcoholic drinks per week	Participants report the average quantity of all types of alcoholic beverages consumed each week	Liu M (2018)		335 394	11 887 865	European
ebi-a-GCST90016564	IBS	Genome Wide Association Study (GWAS) Data set Identifier Related to Irritable Bowel Syndrome (IBS)	Eijsbouts C (2021)		486 601	9 739 966	European

BMI – body mass index, SNP – Single Nucleotide Polymorphism

### Ethics

We sourced all data from publicly accessible databases that relevant ethics committees had previously approved. Ethical clearance for this study was waived, as no primary data collection was conducted. Each participant provided written informed consent in their respective studies, which received approval from their institutional ethics review boards.

### Data preprocessing

Variants reaching genome-wide significance (*P* < 5 × 10^−6^) were clustered to select instrumental variables from the GWAS. An independence threshold of 10 000 kilobase pairs (kb) (where 1 kb equals 1000 base pairs) was applied, with r^2^ (the squared correlation coefficient, which measures the degree of linkage disequilibrium between two genetic markers) set below 0.001. With the use of PhenoScanner, which provides phenotypic data for SNPs, we attempted to minimise the effect of confounding [3,15]. F-statistics were computed to assess the presence of weak IVs, confirming that all instruments were sufficiently strong (F-statistics >10) [16].

For each outcome, independent IVs were selected, with palindromic SNPs excluded. The exposure and outcome data sets were aligned using a harmonisation function. The causal link between exposure and outcome was evaluated by calculating the gene-outcome to gene-exposure association ratio from GWAS data. The inverse-variance weighted (IVW) method was applied to meta-analyse SNP effects. Cochran's Q-test determined heterogeneity; a random-effects IVW model was used when heterogeneity was present, otherwise, a fixed-effects model was applied. Additional methods included: the weighted median estimator (WME) method for reliable estimates when valid SNPs constituted more than 50% of the weight; the penalised weighted median (PWM) method to adjust for genetic variation in cases of heterogeneity; and the maximum likelihood (ML) method to increase observational data likelihood using a phylogenetic tree approach [17].

### Sensitivity analysis

The MR analysis results were tested in different ways to ensure robustness. Since eliminating horizontal pleiotropy increases the reliability of the results and eliminating vertical pleiotropy may distort causal estimates, we tested only for horizontal pleiotropy [18]. We assessed horizontal pleiotropy of individual SNP effects using Cochran’s Q-statistic within IVW and MR-Egger frameworks. MR-Egger analysis differs from IVW by using the intercept term to assess average pleiotropy among instruments, allowing estimation even if all SNPs are invalid under the INSIDE assumptions [19]. A leave-one-out sensitivity analysis was performed to identify instruments that might impact MR outcomes. Heterogeneity was assessed by visually inspecting MR funnel plots and quantified using *P*-values from the Cochran Q-test.

### Multivariate Mendelian randomisation

The TwoSampleMR package aligned effect estimates and sizes. The MVMR analysis then filtered instrumental variables for different exposure factors to assess the causal relationship between eight exposures and depression. Then, ORs were calculated as before.

### Statistics

Our MR study used the TwoSampleMR package. All statistical analyses and visualisations were performed in R Studio using R (version 4.1.0, Auckland, New Zealand), with a significance threshold of *P* < 0.05.

## RESULTS

### Seven exposure factors had a significant causal relationship with IBS

**Figure 2:**
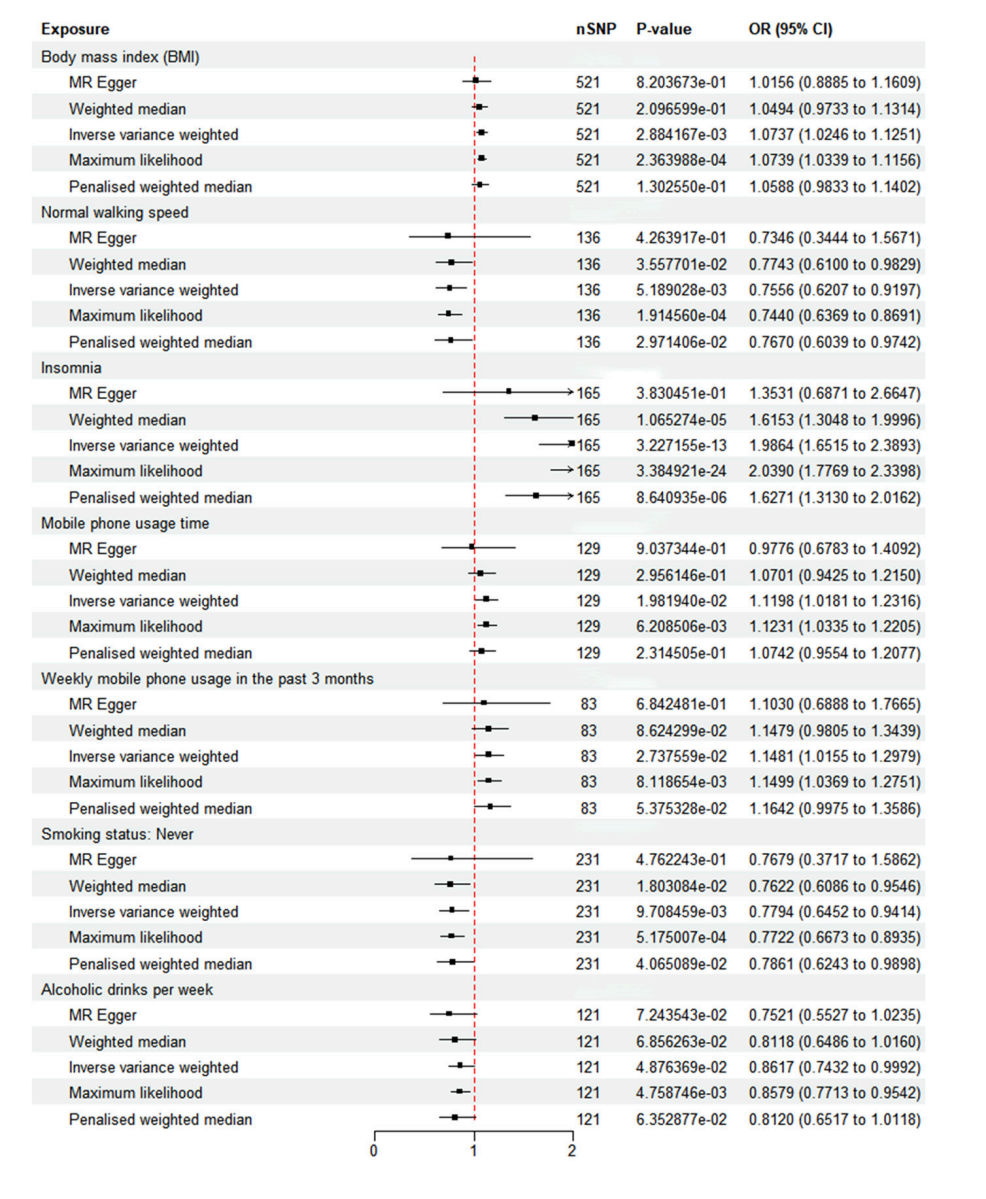
The forest plot illustrates the causal relationship between exposure factors and IBS, as determined using the IVW MR method. IBS – irritable bowel syndrome, IVW – inverse-variance weighted, MR – Mendelian randomisation.

After screening, 521 IVs for body mass index (BMI), 136 for usual walking speed, 165 for insomnia, 129 for duration of mobile phone use, 83 for weekly mobile phone usage time in the past three months, 231 for smoking status (never), and 121 for weekly alcohol consumption were selected (Table S1–7 in the **Online Supplementary Document**). The IVW method indicated a causal link between BMI (OR = 1.074; 95% CI = 1.025–1.125, *P* = 0.031), usual walking speed (OR = 0.756; 95% CI = 0.621–0.920, *P* < 0.001), insomnia (OR = 1.986; 95% CI = 1.652–2.389, *P* < 0.001), duration of mobile phone use (OR = 1.120; 95% CI = 1.018–1.232, *P* = 0.021), weekly mobile phone use over the last three months (OR = 1.148; 95% CI = 1.016–1.298, *P* = 0.021), smoking status (OR = 0.779; 95% CI = 0.645–0.941, *P* < 0.001), and weekly alcohol drink intake (OR = 0.862; 95% CI = 0.743–0.999, *P* = 0.015) and IBS (**Figure 2**). Positive slopes in these analyses indicated risk factors, such as BMI, insomnia, and frequent mobile phone use. In contrast, usual walking speed, non-smoking status, and weekly alcohol intake presented as protective factors for IBS (Figure S1, Panels A–G, in the **Online Supplementary Document**). In addition, to distinguish between weekly consumption of alcoholic beverages (moderate drinking) and frequency of alcohol intake (problematic drinking), we also compared the causal association between the two for IBS (Figure S2 in the **Online Supplementary Document**). Forest plots were aligned with leave-one-out analysis results, confirming reliability (Figure S3, Panels A–G, in the **Online Supplementary Document**), with no significant bias observed in leave-one-out findings (Figure S4, Panels A–G, in the **Online Supplementary Document**). Funnel plots exhibited symmetrical sample distribution around the IVW line, supporting the consistency of UVMR results with Mendelian randomisation assumptions (Figure S5, Panels A–G, in the **Online Supplementary Document**).

This study found heterogeneity among exposure factors, including BMI, usual walking speed, insomnia, mobile phone usage duration, weekly mobile phone use over the last three months, non-smoking status, and weekly alcohol consumption (**Table 2**). To account for potential confounding effects among different exposures, False Discovery Rate (FDR) correction method was applied. As presented in the table, all detected associations retained statistical significance (*P* < 0.05) following FDR correction, suggesting the robustness of our observational findings (Table S8 in the **Online Supplementary Document**).

**Table 2:** Tests for heterogeneity and horizontal pleiotropy of MR analyses

Exposure	Outcome	Heterogeneity		Horizontal pleiotropy	
		**MR-egger (Q)**	***P*-value**	**Egger intercept**	***P*-value**
BMI	IBS	814.6358	1.75E-15	0.0011	0.3848
Usual walking pace	IBS	231.4073	3.56E-07	0.0003	0.9401
Insomnia	IBS	320.4401	2.44E-12	0.0037	0.2502
Length of mobile phone use	IBS	176.1259	2.58E-03	0.0026	0.4523
Weekly usage of mobile phone in last 3 mo	IBS	122.8898	1.86E-03	0.0007	0.8635
Smoking status: Never	IBS	410.4722	1.89E-12	0.0001	0.9669
Alcoholic drinks per week	IBS	243.9560	1.24E-10	0.0022	0.3256
IBS	BMI	182.5108	7.34E-18	−0.0013	0.6327
IBS	Usual walking pace	87.9857	2.72E-04	0.0009	0.4343
IBS	Insomnia	147.5944	2.55E-12	0.0025	0.0940
IBS	Length of mobile phone use	95.9092	3.35E-05	0.0009	0.6819
IBS	Weekly usage of mobile phone in last 3 mo	104.1551	3.27E-06	0.0036	0.1539
IBS	Smoking status: Never	293.8572	4.33E-35	−0.0001	0.9373
IBS	Alcoholic drinks per week	100.8836	1.49E-06	0.0004	0.8217

BMI – body mass index, IBS – irritable bowel syndrome, MR – Mendelian randomisation

Vertical pleiotropy can potentially influence the results of MR analyses, which prompted us to perform supplementary MR-PRESSO analyses to strengthen our causal inferences. Our results revealed that all exposures, except for ‘Length of mobile phone use’, maintained statistical significance after outlier correction, confirming the relative robustness of our findings. For mobile phone use duration, the lack of significance after adjustment suggests potential vertical pleiotropy effects, highlighting the need for future cohort studies to further validate these causal relationships (Table S9 in the **Online Supplementary Document**). Power calculations revealed effect size detection probabilities ranging from 0.741 to 0.997 across all exposures, indicating that most analyses possessed sufficient statistical power to reliably detect true causal effects (Table S10 in the **Online Supplementary Document**). Heterogeneity test results showed *P* values less than 0.05 for both Cochran's Q and IVW, suggesting that although significant heterogeneity was present, the findings remained statistically significant and robust against this variation (**Figure 2**).

### Reverse MR analysis reveals no causal link between IBS and exposure factors, except for insomnia

Given the potential bidirectional effects between exposure factors and outcomes, a reverse MR analysis was conducted to validate the causal link between IBS and seven behavioural factors. The findings showed no significant causality between IBS and these factors, except for insomnia, thus reinforcing the connection between behavioural factors and IBS risk (Figure S6 in the **Online Supplementary Document**). A causal relationship between IBS and insomnia was revealed through the IVW method, with an OR of 1.0645 (95% CI = 1.0413–1.0881). Sensitivity analyses corroborated this finding, with the weighted median showing an OR of 1.0372 (95% CI = 1.0163–1.0585), the maximum likelihood showing an OR of 1.0717 (95% CI = 1.0571–1.0865), and the penalised weighted median showing an OR of 1.0361 (95% CI = 1.0162–1.0565).

### Direct impact of weekly mobile phone use, insomnia, and weekly alcohol intake on IBS in the presence of multiple factors

**Figure 3:**
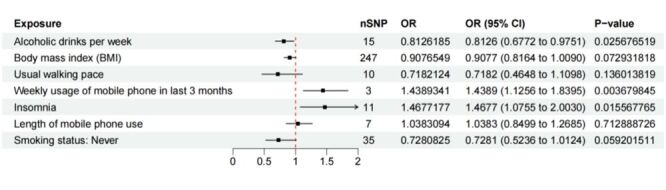
The forest plot of the MVMR analysis.

After screening, 328 SNPs were selected as instrumental variables (IVs) for the MVMR analysis, covering seven exposure factors BMI, usual walking speed, insomnia, mobile phone use duration, weekly mobile phone usage time in the past three months, non-smoking status, and weekly alcohol intake. The MVMR analysis revealed that weekly mobile phone usage time in the past three months (OR = 1.439; 95% CI = 1.126–1.840, *P* = 0.0037) and insomnia (OR = 1.468; 95% CI = 1.076–2.003, *P* = 0.0156) were risk factors, while weekly alcohol intake (OR = 0.813; 95% CI = 0.677–0.975, *P* = 0.0257) acted as a protective factor. Furthermore, in the combined analysis, weekly mobile phone use, insomnia, and weekly alcohol consumption showed a direct influence on IBS (**Figure 3**). We also included common confounding factors in daily life in the multivariate MR analysis, and the conclusion still holds true (Figure S7 in the **Online Supplementary Document**). The ORs for the other factors were as follows BMI (OR = 0.9077; 95% CI = 0.8164–1.0090, *P* = 0.0729), usual walking pace (OR = 0.7182; 95% CI = 0.4648 to–1.1098, *P* = 0.1360), smoking status (never) (OR = 0.7281; 95% CI = 0.5236–1.0124, *P* = 0.0592), and length of mobile phone use (OR = 1.0383; 95% CI = 0.8499–1.2685, *P* = 0.7129).

## DISCUSSION

This study used univariable and MVMR to explore causal links between various behavioural factors and IBS. Our findings indicated significant causal associations between IBS and BMI, usual walking pace, weekly mobile phone usage in the past three months, never smoking status, and weekly alcohol consumption. Multivariate MR analysis also found that there is still a potential association between weekly alcohol consumption, mobile phone usage in the past three months, and IBS. Additionally, reverse MR analysis suggested that IBS might lead to insomnia.

Previous observational research has produced varied results when examining the link between physical activity and the risk of IBS. Some findings indicate that physical activity might enhance bowel motility and decrease inflammation, lowering the risk of IBS. However, other studies failed to find significant associations, possibly due to small sample sizes, different study designs, or inadequate control of confounding factors [20,21]. Results from our univariable Mendelian randomisation (UVMR) analysis indicated a significant inverse association between usual walking pace and IBS risk (OR = 0.756; 95% CI = 0.621–0.920, *P* = 0.0052), providing more robust causal evidence and supporting the importance of physical activity in preventing IBS. This finding aligns with those of several previous observational studies indicating that physical activity may positively impact gut health through various mechanisms. Regular walking may protect against IBS via multiple physiological mechanisms. First, as a low-intensity aerobic exercise, walking can promote bowel motility, improve bowel function, and reduce symptoms such as constipation and bloating [22]. Walking can also increase intestinal blood flow, enhancing the nutrient supply to intestinal cells and waste elimination, thus improving gut health [23]. Additionally, walking may decrease both systemic and localised inflammatory responses, thereby reducing IBS risk. Studies suggest that physical activity can lower levels of pro-inflammatory cytokines, such as tumour necrosis factor-alpha (TNF-α) and interleukin-6 (IL-6), while increasing anti-inflammatory cytokines like interleukin-10 (IL-10) [24,25]. Additionally, walking can improve mental health, thereby decreasing IBS risk. Research indicates that physical activity can alleviate stress and anxiety, improve mood, and consequently alleviate IBS symptoms [26]. Walking, as a simple and accessible exercise, can enhance mental well-being by increasing endorphin secretion and promoting happiness and satisfaction [27,28]. These findings provide scientific evidence for applying physical activity in IBS prevention and management. Additional studies are required to confirm these mechanisms and investigate new intervention strategies.

Previous studies indicated that prolonged smartphone usage is associated with various health issues, including sleep disorders, anxiety, and depression [29–31]. In this study, we found that an increase in weekly smartphone usage significantly raises the risk of developing IBS. This association can be attributed to several factors. First, extended smartphone use may lead to insomnia, primarily manifested as delayed bedtimes and reduced overall sleep duration [32]. Possible mechanisms include time displacement, where screen time replaces time that could be used for sleep, psychological stimulation from media content, and blue light emitted by devices, which may affect melatonin levels and disrupt circadian rhythms, potentially resulting in gut microbiota imbalance that exacerbates IBS symptoms [33–35]. Second, excessive smartphone usage may contribute to increased psychological stress and anxiety [36], both closely linked to the onset of IBS. Prolonged smartphone use often involves constant notifications and updates, creating a sense of urgency and fear of missing out, which can keep individuals in a state of anxiety and stress [37]. Additionally, constant exposure to social media can foster negative social comparisons and feelings of inadequacy, further amplifying anxiety. This psychological pressure might activate the hypothalamic-pituitary-adrenal (HPA) axis, leading to elevated cortisol levels, which can trigger inflammatory responses and alter neurotransmitter systems, such as serotonin, crucial for gut function and mood regulation [38,39]. The interplay between stress hormones and neurotransmitters may increase intestinal permeability and contribute to the worsening of IBS symptoms [40,41]. Therefore, we recommend that the public use smartphones in moderation and avoid prolonged screen time to protect mental health and gut function, ultimately reducing the risk of IBS.

Besides, our MVMR results showed the bidirectional relationship between insomnia and IBS, indicating that insomnia significantly increases the risk of IBS, while IBS also significantly increases the risk of insomnia, suggesting that insomnia and IBS promote and exacerbate each other. A retrospective study showed that the prevalence of insomnia in IBS was 41.2% and confirmed that insomnia was an independent risk factor for IBS (OR = 1.81; 95% CI = 1.44–2.27) [42]. In addition, treating sleep difficulties in IBS patients has been shown to improve IBS symptoms [43,44]. Mechanistically, insomnia leads to the disruption of biological rhythms, which affects the integrity of the intestinal barrier through the β-catenin-MMP pathway, and causes overactivation of the sympathetic nervous system [35], further aggravating intestinal dysfunction. Some studies have also shown that sleep disorders may lead to increased levels of inflammation in the body [45], which is related to the pathological mechanism of IBS. Conversely, IBS can also cause insomnia. Previous studies have indicated that sleep disorders are associated with IBS-related pain, distress, and poor IBS-related quality of life [46]. On average, IBS patients sleep longer but rest less well than healthy controls [47]. Mechanistically, symptoms such as abdominal pain, bloating, and intestinal discomfort often associated with IBS can disturb sleep, making it difficult to fall asleep or stay asleep. Nocturnal abdominal pain and frequent nocturnal bowel movements significantly affect sleep quality. Additionally, the intestinal flora of IBS patients is often altered, which may further affect mental state, sleep quality, and the host's circadian rhythm, exacerbating insomnia [47]. Therefore, there may be a mutually reinforcing relationship between insomnia and IBS, suggesting that when addressing patients with insomnia and IBS, it is essential to consider their mutual influence and co-treatment, intervening simultaneously to promote faster recovery.

Smoking and alcohol consumption, as notable behavioural habits, have been widely studied for their associations with IBS. Research has indicated that smoking may elevate the risk of developing IBS [48] Certain studies suggest that moderate alcohol consumption may offer a protective effect against adverse cardiovascular events [49]. Ho et al. further showed that non-smoking status is strongly linked to a reduced incidence of IBS in the general population [50]. Our MR analysis indicated that non-smoking status significantly lowers IBS risk. The result is consistent with some previous research results, further supporting the correlation between smoking and IBS risk. Mechanistically, nicotine and other harmful substances in cigarettes may affect the gastrointestinal nervous system, change intestinal motility and lead to abnormal defecation [51]. Interestingly, our Mendelian randomisation analysis revealed differential effects of moderate and excessive alcohol consumption on IBS (Figure S2 in the **Online Supplementary Document**). The results showed that while moderate drinking may have a protective effect against IBS, excessive drinking may exacerbate its progression. Previous studies have shown that moderate alcohol consumption can help relieve stress and anxiety by increasing the activity of gamma-aminobutyric acid (GABA) in the brain, thereby improving mood and reducing IBS symptoms [52]. Besides, Moderate alcohol consumption, especially the consumption of polyphenolic compounds such as resveratrol in red wine, has anti-inflammatory effects and can reduce oxidative stress [53]. This may alleviate IBS symptoms by decreasing the production of free radicals and inflammatory mediators like TNF-α and IL-1, along with pro-inflammatory enzymes such as iNOS and COX-2, and reducing activity in inflammatory signalling pathways like NF-kB [54]. However, excessive alcohol consumption can lead to increased gut permeability, inflammation, and disruption of the gut microbiota, thereby exacerbating IBS symptoms. Therefore, the results indicated that different patterns of alcohol consumption might be important factors affecting IBS symptoms, and the dose-dependent dual nature of alcohol's impact on health. These findings highlight the need for randomised controlled trials to better understand the effects of low alcohol intake on IBS symptoms.

Besides, previous studies have shown that socioeconomic status, diet and physical health can influence IBS symptoms and management. For example, one cohort study showed that the prevalence of IBS was significantly higher among people with lower household income [55]. In addition, studies have shown that eating smaller meals and reducing alcohol and caffeine intake are beneficial for the relief of IBS [56]. In addition, moderate increase in physical activity can improve gastrointestinal symptoms of IBS. This long-term follow-up showed that symptom improvement was more significant about 5 years after participating in an intervention to increase physical activity [57]. Therefore, we conducted multivariate Mendelian randomisation analysis by incorporating factors such as socioeconomic status. The results demonstrated that our conclusions remained robust even after adjusting for these additional factors, supporting the validity and reliability of our findings (Figure S7 in the **Online Supplementary Document**). Nevertheless, it should be acknowledged that it is challenging to completely rule out the influence of all aspects. Although the adjusted causal relationships between BMI, usual walking pace, never smoking status, and mobile phone use and IBS did not reach statistical significance in this study, these factors still warrant further investigation in future research.

### Limitations

The data used in our research primarily originated from patients of European descent, which necessitates caution when generalising these findings to other populations. This limitation can be attributed to three key factors: genetic polymorphisms across populations, significant differences in traditional dietary patterns and alcohol consumption habits among ethnic groups, and cultural variations in the perception and expression of IBS symptoms. Previous studies have shown that the ALDH2*2 variant, which affects alcohol metabolism, exhibits marked frequency differences between East Asian and European populations [58]. Additionally, research indicates that dietary patterns and alcohol consumption behaviours vary significantly across cultures, with distinct preferences for different types of alcoholic beverages [59,60]. Furthermore, cultural backgrounds influence how individuals perceive and report gastrointestinal symptoms, potentially affecting IBS diagnosis and management [61]. Therefore, future research utilising GWAS data from diverse populations is essential to validate the generalisability of our findings.

Although we tried to control for various confounding factors, some potential confounders may still affect our assessment results. Due to the differing manifestations of IBS across different sexes and age groups and the limited disclosure of data, we could not conduct more detailed stratified analyses.

While our MR analyses suggest an association between moderate alcohol consumption and IBS risk, it is crucial to recognise the inherent limitations of MR studies in establishing causality. These limitations include potential residual pleiotropy and the inability to fully account for nonlinear relationships. Our findings should be interpreted as hypothesis-generating rather than definitive causal evidence. The observed associations, while intriguing, require validation through prospective clinical studies and careful consideration of potential biological mechanisms.

## CONCLUSIONS

Body mass index, insomnia, duration of mobile phone use, and weekly mobile phone use in the past three months emerged as risk factors. Conversely, weekly alcohol consumption, usual walking speed, and non-smoking status were identified as protective factors. Additionally, when multiple factors were analysed together, weekly mobile phone use, insomnia, and weekly alcohol intake showed a direct association with IBS.

## Additional material


Online Supplementary Document
