# Supplementary material for: Unravelling behavioural contributions to IBS risk: evidence from univariate and multivariate Mendelian randomisation
Source: J Glob Health. 2025 Apr 11;15:04112. doi: 10.7189/jogh.15.04112 (PMC11987576; doi:10.7189/jogh.15.04112)

Supplement to: Hongyu C, Yumin Z, Jiayi Z. Unravelling behavioural contributions to IBS risk: evidence from univariate and multivariate Mendelian randomisation. J Glob Health. 2025;15:04112.

Table S1. The Single nucleotide polymorphisms (SNPs) list of ukb-a-248_BMI.

Table S2. The SNPs list of ukb-a-513_Usual walking pace.

Table S3. The SNPs list of ukb-b-3957_Sleeplessness / insomnia.

Table S4. The SNPs list of ukb-b-4094_Length of mobile phone use.

Table S5. The SNPs list of ukb-b-17999_Weekly usage of mobile phone in last 3 months.

Table S6. The SNPs list of ukb-d-20116_0_Smoking status: Never.

Table S7. The SNPs list of ieu-b-73_Alcoholic drinks per week.

Table S8. The results of the FDR correction

Table S9. MR-PRESSO analysis with IBS as outcome

Table S10. Statistical Power Estimates for Causal Inference in Mendelian Randomization

Figure S1. A scatter plot of the MR analysis. Scatter plots are presented in (Panels A﹣G) to illustrate the causal association between depression and BMI (Panel A), walking speed (Panel B), insomnia (Panel C), length of cell phone use (Panel D), weekly mobile phone usage in the last three months (Panel E), smoking status: never (Panel F), and alcohol consumption per week (Panel G). Different MR algorithms are represented by coloured lines. Positive slopes indicate risk factors, while negative slopes indicate protective factors. BMi ﹣ body mass index, MR ﹣ Mendelian randomisation.

Figure S2. Mendelian randomization analysis of moderate drinking and problematic drinking

Figure S3. Forest plot illustrating the relationship between exposures and depression using IVW MR values. (A) Body Mass Index; (B) Walking speed; (C) Insomnia; (D) Mobile phone usage; (E) Weekly usage in the last three months; (F) Smoking status: Never; (G) Number of alcoholic drinks per week. On the left, solid points indicate a decrease, while on the right, solid points indicate an increase.

Figure S4. Leave-one-out MR analysis. (A) Body mass index (B); (B) Usual walking pace; (C) Insomnia; (D) The number of hours spent on mobile phones during the last three months; (E) Weekly usage of mobile phones; (F) Smoking status: Never; (G)

Alcoholic drinks per week.

Figure S5. MR analysis funnel plot. (A-G) Funnel plots showing the overall heterogeneity of MR analysis for the effects of BMI (A), regular walking pace (B), insomnia (C), length of mobile phone use (D), use of mobile phones each week in the past three months (E), smoking status (F), and alcohol consumption per week (G).

Figure S6. The forest plot illustrated the studies that assessed the causal impact of IBS on seven behavioral factors, using the values derived from the IVW MR method.

Figure S7. Multivariate Mendelian randomization analysis considering other common confounding factors

Table S8. The results of the FDR correction. After FDR correction, there is still a significant causal association between all exposures and outcomes.

Exposure	***P*-value**	**Rank**	**q = p * (7 / rank)**
usual walking speed	< 0.001	1	< 0.007
insomnia	< 0.001	2	< 0.0035
smoking status	< 0.001	3	< 0.0023
weekly alcohol intake	0.015	4	0.0263
duration of mobile phone use	0.021	5	0.0294
weekly mobile phone use over the last three months	0.021	6	0.0245
BMI	0.031	7	0.031

Table S9. MR-PRESSO analysis with IBS as outcome.

GWAS ID	Exposure	Number of SNPs	IVW P-value	MR-PRESSO P-value	Outlier-corrected P-value
ukb-a-248	BMI	521	0.031	0.0036	0.0029
ukb-a-513	Usual walking pace	136	< 0.001	0.0098	0.0159
ukb-b-3957	Insomnia	165	< 0.001	< 0.001	< 0.001
ukb-b-4094	Length of mobile phone use	129	0.021	0.0517	N/A
ukb-b-17999	Weekly usage of mobile phone in last 3 months	83	0.021	0.0309	0.0224
ukb-d-20116_0	Smoking status: Never	231	< 0.001	0.0062	0.0680
ieu-b-73	Alcoholic drinks per week	121	0.015	0.0707	0.0324

Table S10. Statistical Power Estimates for Causal Inference in Mendelian Randomization.

Trait	Sample size	OR	Power
BMI	336107	0.071078572	0.741
Usual walking pace	335349	-0.28029124	0.99
Insomnia	462341	0.686323896	0.955
Length of mobile phone use	456972	0.11314978	0.928
Weekly usage of mobile phone in last 3 months	386626	0.138066952	0.775
Smoking status: Never	359706	-0.2492779	0.997
Alcoholic drinks per week	335394	-0.14881371	0.778

Figure S1. A scatter plot of the MR analysis. Scatter plots are presented in (Panels A – G) to illustrate the causal association between depression and BMI (Panel A), walking speed (Panel B), insomnia (Panel C), length of cell phone use (Panel D), weekly mobile phone usage in the last three months (Panel E), smoking status: never (Panel F), and alcohol consumption per week (Panel G). Different MR algorithms are represented by coloured lines. Positive slopes indicate risk factors, while negative slopes indicate protective factors. BMi – body mass index, MR – Mendelian randomisation.

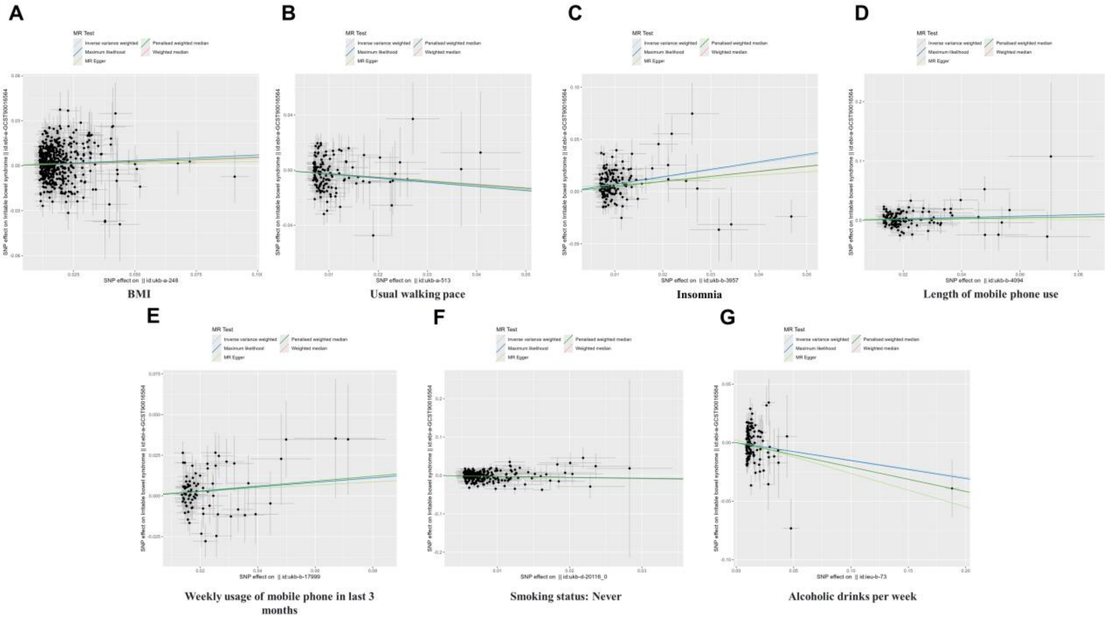

Figure S2. Mendelian randomization analysis of moderate drinking and problematic drinking.

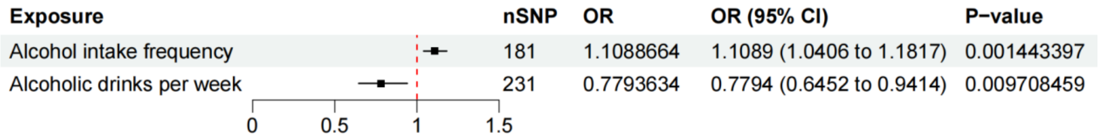

Figure S3. Forest plot illustrating the relationship between exposures and depression using IVW MR values. (A) Body Mass Index; (B) Walking speed; (C) Insomnia; (D) Mobile phone usage; (E) Weekly usage in the last three months; (F) Smoking status: Never; (G) Number of alcoholic drinks per week. On the left, solid points indicate a decrease, while on the right, solid points indicate an increase.

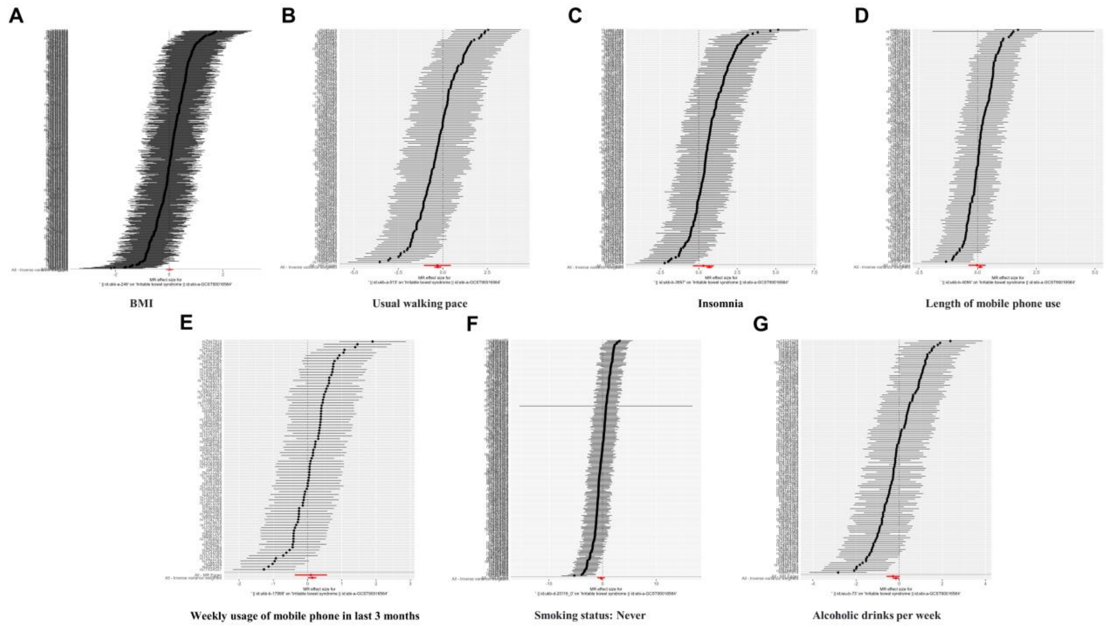

Figure S4. Leave-one-out MR analysis. (A) Body mass index (B); (B) Usual walking pace; (C) Insomnia; (D) The number of hours spent on mobile phones during the last three months; (E) Weekly usage of mobile phones; (F) Smoking status: Never; (G) Alcoholic drinks per week.

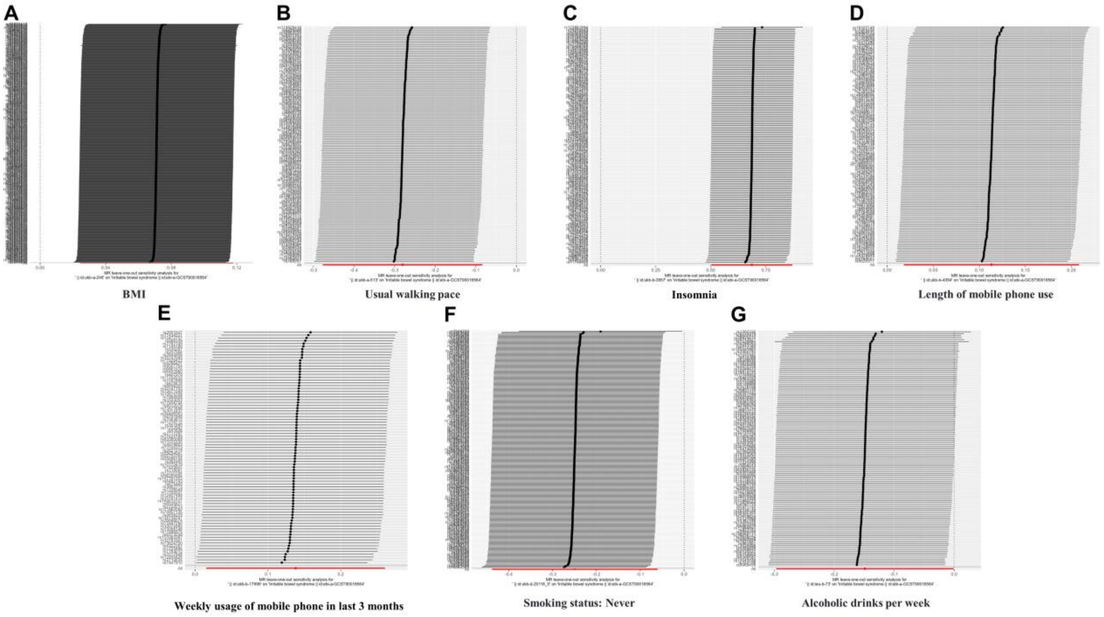

Figure S5. MR analysis funnel plot. (A-G) Funnel plots showing the overall heterogeneity of MR analysis for the effects of BMI (A), regular walking pace (B), insomnia (C), length of mobile phone use (D), use of mobile phones each week in the past three months (E), smoking status (F), and alcohol consumption per week (G).

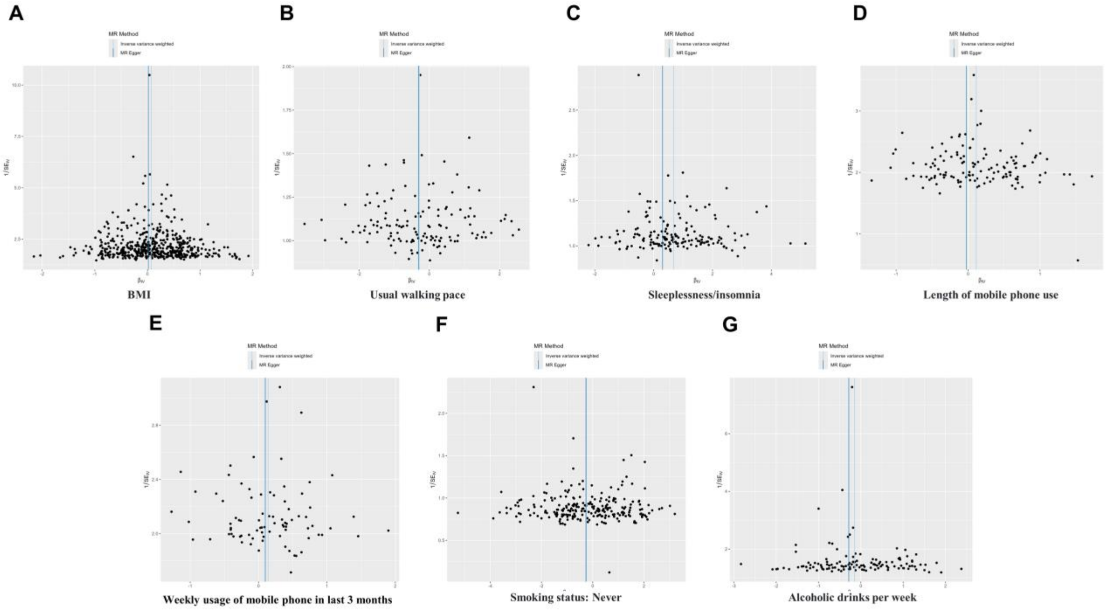

Figure S6. The forest plot illustrated the studies that assessed the causal impact of IBS on seven behavioral factors, using the values derived from the IVW MR method.

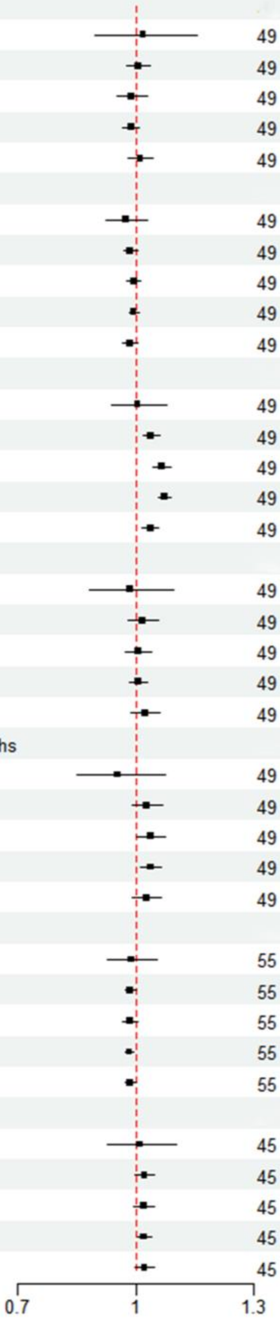

Outcome	nSNP	OR	OR (95% CI)
Body mass index (BMI)			
MR Egger	49	1.0177062	1.0177 (0.8968 to 1.1549)
Weighted median	49	1.0055616	1.0056 (0.9749 to 1.0372)
Inverse variance weighted	49	0.9880885	0.9881 (0.9508 to 1.0268)
Maximum likelihood	49	0.9877075	0.9877 (0.9668 to 1.0091)
Penalised weighted median	49	1.0108790	1.0109 (0.9796 to 1.0432)
Normal walking speed			
MR Egger	49	0.9734249	0.9734 (0.9221 to 1.0277)
Weighted median	49	0.9850575	0.9851 (0.9673 to 1.0031)
Inverse variance weighted	49	0.9938500	0.9938 (0.9775 to 1.0104)
Maximum likelihood	49	0.9936763	0.9937 (0.9812 to 1.0063)
Penalised weighted median	49	0.9847885	0.9848 (0.9661 to 1.0039)
Insomnia			
MR Egger	49	1.0040301	1.0040 (0.9358 to 1.0773)
Weighted median	49	1.0371835	1.0372 (1.0163 to 1.0585)
Inverse variance weighted	49	1.0644555	1.0645 (1.0413 to 1.0881)
Maximum likelihood	49	1.0716599	1.0717 (1.0571 to 1.0865)
Penalised weighted median	49	1.0361308	1.0361 (1.0162 to 1.0565)
Mobile phone usage time			
MR Egger	49	0.9833652	0.9834 (0.8822 to 1.0961)
Weighted median	49	1.0174739	1.0175 (0.9804 to 1.0559)
Inverse variance weighted	49	1.0049851	1.0050 (0.9724 to 1.0386)
Maximum likelihood	49	1.0053784	1.0054 (0.9813 to 1.0301)
Penalised weighted median	49	1.0235086	1.0235 (0.9873 to 1.0610)
Weekly mobile phone usage in the past 3 months			
MR Egger	49	0.9537545	0.9538 (0.8482 to 1.0725)
Weighted median	49	1.0273663	1.0274 (0.9895 to 1.0667)
Inverse variance weighted	49	1.0358645	1.0359 (0.9989 to 1.0742)
Maximum likelihood	49	1.0374918	1.0375 (1.0115 to 1.0642)
Penalised weighted median	49	1.0253632	1.0254 (0.9885 to 1.0637)
Smoking status: Never			
MR Egger	55	0.9871234	0.9871 (0.9251 to 1.0533)
Weighted median	55	0.9858087	0.9858 (0.9723 to 0.9996)
Inverse variance weighted	55	0.9846808	0.9847 (0.9644 to 1.0053)
Maximum likelihood	55	0.9826222	0.9826 (0.9728 to 0.9925)
Penalised weighted median	55	0.9854876	0.9855 (0.9714 to 0.9998)
Alcoholic drinks per week			
MR Egger	45	1.0101522	1.0102 (0.9264 to 1.1015)
Weighted median	45	1.0213133	1.0213 (0.9961 to 1.0471)
Inverse variance weighted	45	1.0198772	1.0199 (0.9948 to 1.0456)
Maximum likelihood	45	1.0208438	1.0208 (1.0034 to 1.0386)
Penalised weighted median	45	1.0212673	1.0213 (0.9960 to 1.0472)

Figure S7. Multivariate Mendelian randomization analysis considering other common confounding factors.

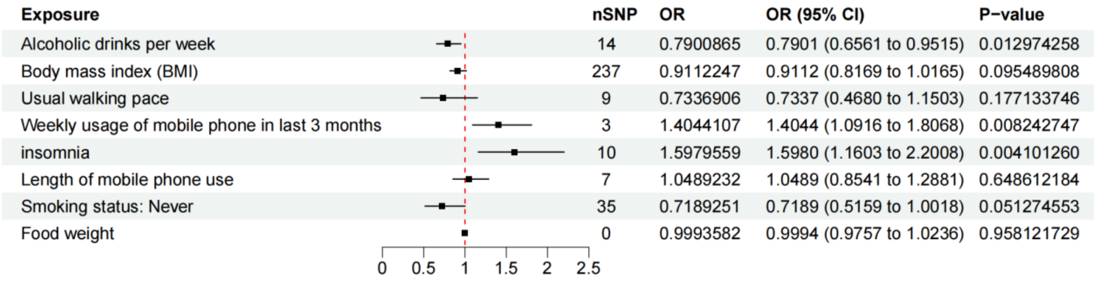

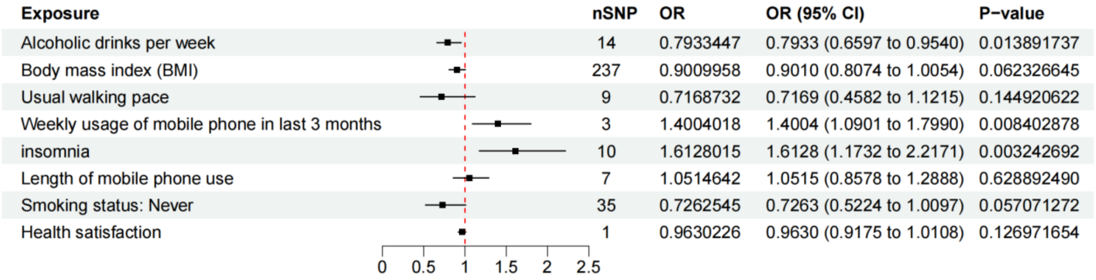

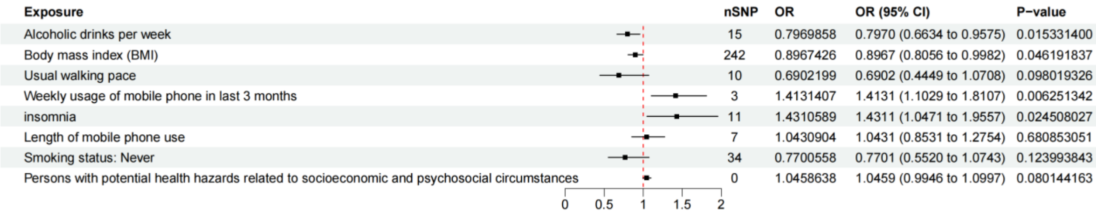

Supplement: Online Supplementary Document [file jogh-15-04112-s001.pdf]
